# The utilization of social networking sites, their perceived benefits and their potential for improving the study habits of nursing students in five countries

**DOI:** 10.1186/s12912-020-00447-5

**Published:** 2020-06-15

**Authors:** Glenn Ford D. Valdez, Arcalyd Rose R. Cayaban, Sadeq Al-Fayyadh, Mehmet Korkmaz, Samira Obeid, Cheryl Lyn A. Sanchez, Muna B. Ajzoon, Howieda Fouly, Jonas P. Cruz

**Affiliations:** 1 Research & Community Services Coordinator, Oman College of Health Sciences, Salalah, Dhofar Oman; 2 Sultan Qaboos University, Muscat, Oman; 3 University of Bagdad, Baghdad, Iraq; 4 Ondokuz Mayıs Üniversitesi, Samsun, Turkey; 5 Max Stern Yezreel Valley College, Jezreel Valley, Israel; 6 School of Nursing, Northern Luzon Adventist College, Pangasinan, the Philippines; 7 Oman College of Health Sciences, Salalah, Dhofar Oman; 8 Assiut University, Assiut, Egypt; 9 Shaqra University, Shaqra, Saudi Arabia

**Keywords:** Social networking sites, Nursing students, Study habits, SNS benefits

## Abstract

**Background:**

The abundance of easy and accessible information and the rapid development of social networking sites (SNSs) have proven that the world is small and within reach. The great implication of this interconnectivity is attributable to the change in the learning and sharing environment, which for the most part is something that classrooms are lacking. Considering the potential implications of SNSs in nursing education reveals the benefits of SNSs in allowing students to communicate and interact with a wider audience and beyond the classroom. The aim of this study is to identify the extent of SNS utilization, the perceived benefits of SNSs and the potential of SNSs for improving the study habits of nursing students in five countries (Israel, Iraq, Oman, the Philippines and Turkey).

**Methods:**

This study is a quantitative cross-sectional study that determined the relationship between the utilization of SNSs, the perceived benefits of SNSs, and the potential of SNSs for improving the study habits of nursing students in the five participating countries (Israel, Iraq, Oman, the Philippines, and Turkey). This paper is based on carefully analysing the survey responses of a sample of 1137 students from an online hosting site. The online instrument focuses on the extent of the utilization and benefits of SNSs according to their accessibility, usability, efficiency and reliability.

**Results:**

Based on the Pearson correlation coefficient (r) our findings, reveal a significant positive correlation between the extent of a possible improvement in study habits and the extent of SNS utilization in terms of the four domains, namely, accessibility (r = 0.246), usability (r = 0.377), reliability (r = 0.287) and efficiency (r = 0.387).

**Conclusion:**

It can be concluded that there is a significant positive correlation between students’ study habits and the extent of SNS utilization, meaning that the more students devote themselves to their study habits, the higher the level of SNS utilization. The use of SNSs by nursing students has positive and negative implications, and there is greater potential for further improving approaches to nursing education through the adaptation of curricula based on the proper utilization of SNSs.

## Background

In today’s generation, the rapid and ever-changing advances in technology and interconnectivity through networking has dramatically influenced the culture of learning and knowledge acquisition. The abundance of easy and accessible information and the rapid development of social networking sites (SNSs) have proven that the world is small and within reach. The great implication of this interconnectivity is attributable to the change in the learning and sharing environment, which for the most the part is something that classrooms are lacking. Additionally, social media in nursing education have shown great potential for influencing students’ study habits [1]. Online SNSs (e.g., Facebook, Myspace, Flicker, Twitter, and YouTube) have emerged as the fastest means of exchanging personal and professional information among college students [2]. SNS utilization is defined as the utilization of information networks as a form of communication widely used for several purposes. SNSs are used to interact with users and to generate content, and in recent years, they have seen expansion with regard to creating and maintaining relationships between people [3]. The issues related to SNSs are unlimited, but there is growing research on the use of social media as learning tools in higher education [4]. SNSs function like an online community of web users, depending on the website, and many of online SNSs are based on a shared interest. Once accessed, users may begin to socialize. This socialization may include reading the profile pages of other members and possibly even contacting them. The profiles of SNS users vary according to users’ discretion with regard to privacy and their visibility settings [5]. In this age of technological acuity, the world has become too small, and communication has become more efficient than ever. SNSs have played a vital role in forging connections, and Facebook is the most popular SNS in use today. Facebook has become one of the most regularly visited websites among college students, and because of its rise in popularity, the subject of SNSs among students and faculty has been a topic of concern. SNSs are seen as an alternative to social interaction, access to information and face-to-face interaction. SNSs, such as Facebook, seem to provide a ready space where the role conflicts that students and faculty often experience in their relationship with university work, staff, academic conventions, and expectations can be worked out in a backstage area. SNSs, such as Twitter, are utilized as a tool for posting explanations in study groups, for academic advising, and for student education [5]. Many researchers have discussed the broad benefits of SNSs in higher education [6]. Nursing students have identified three proposed reasons for the use of social media to learn through social networking and to socialize with other students, thus establishing professional social networking [7]. First, SNSs also allow communication with students through instant messages. Second, they enable rapid responses to questions asked by students, and they facilitate virtual discussions that make students part of a community. Third, SNSs also allow active, interactive and reflective learning [8]. A study on the use of Facebook for online discussions among distance learners showed that there was more frequent interaction via Facebook compared to the use of a forum, which indicates that Facebook has the potential to be used in online academic discussions [9]. The use of Twitter allowed connections between students, access to external resources, improved learning, and support to access videos, providing opportunities for reflection, flexibility, collaboration, and feedback [10]. The use of a social networking tool called Ning verifies the feasibility and effectiveness of integrating interprofessional education, which most students showed interest in learning more about, and optimizing patient care [11]. The use of social networking platforms is a less expensive way to provide interpersonal education, and it creates the possibility of implementing interprofessional education on a large scale and in the long term [11]. A study identified that most students agree that the use of SNSs, such as Ning, contributed to adding knowledge and increasing their understanding of content [12]. A study considering the potential implications of SNS for nursing education revealed the benefits of SNSs in allowing students to communicate and interact with a wider audience and beyond the classroom [13]. One example is the creation of a research group called the mentor and researcher group (MARG), which creates mentors who use Facebook as a communication platform to promote events and serve as a network to discuss issues and concerns among nursing students [14]. Students realize that Facebook groups can be an innovative method of studying. Facebook has also been described as being useful in promoting learning among peers and teachers [15]. SNSs are widely used among college students and are beneficial to them because they have the ability to gather students from all over the world to mingle in one virtual world [16]. This also means that campuses can now begin to blend the subject areas of classes as well as different campuses. A similar study agreed that students spend, on average, 1–2 h a day on SNSs for educational purposes [17]. In this respect, a study on social networks and learning stated that students listed learning as a top priority when utilizing SNSs [18]. In contrast, other studies say that Facebook leads to lower grades [17]. Students have reported concerns that include time management issues, lack of information and communication technology (ICT) skills and limited technical infrastructure in some higher education institutions [6]. The use of social media has greatly shown an unlimited influence on a student’s general lifestyle. This research was empirically designed to identify the degree of SNS utilization by nursing students, the perceived benefits of SNSs and their potential for improving the study habits of students. This study also seeks to determine the relationship between the utilization of SNSs, their perceived benefits, and their potential for improving the study habits of nursing students in five countries. That is, this study was conducted in five countries: Israel, Iraq, Oman, the Philippines and Turkey. Geographically and demographically, Israel, Iran, Oman and Turkey are homogenous in terms of their settings and cultural background. On the other hand, although it is also part of Asia, the Philippines is more geographically and demographically different in many ways. According to the Internet World Statistics in 2019, the Philippines, Iran and Turkey were among the top 20 counties in the world with regard to the number of Internet users; on the other hand, in Israel and Oman, 3.8 and 2.2% of the population, respectively, are Internet users [19]. There is a scarcity of research that specifically addresses nursing education and the use of SNSs. Therefore, this study generally aims to shed light on the potential of SNSs for improving the study habits of nursing students in these five countries.

### Research questions and hypotheses

This research seeks to answer the following questions: What is the extent to which SNSs are utilized as a means of communication in terms of educational purposes? What social media network is the most helpful for nursing students? What are the perceived benefits of SNSs in terms of accessibility, usability, efficiency and reliability? Is there a significant relationship between the extent of utilization and the perceived benefits of SNSs among nursing students? Does SNS utilization have the potential to improve the study habits of nursing students?

H01: There is no significant relationship between the extent of SNS utilization and the benefits of SNS among nursing students.

HO 2: Using SNSs has no potential to improve the study habits of students.

## Methods

### Study design

This study adopts a quantitative cross-sectional design to determine the relationship between the utilization and perceived benefits of SNSs and their potential for improving the study habits of nursing students in the five participating countries.

### Research settings

This study was conducted in five countries. Country selection and participation involved a voluntary system. This study focused on the utilization and perceived benefits of SNSs and their potential for improving the study habits of regular nursing students in the selected colleges and universities of the participating countries. The study participants consisted of first-year to fifth-year Bachelor of Science in Nursing (BSC) students from the five participating countries.

### Sample and sampling techniques

The sample of respondents of this study constituted a 1200-student cohort selected from all the universities that met the set of inclusion criteria, and based on the online forms returned, 1400 links were forwarded. This purposive sampling technique was used considering the criteria for the population, and a post hoc sample was computed via proportion analysis using a confidence interval of 0.65 and a confidence level of 0.95 for a sample of 1137 students. The **inclusion criteria** were as follows**:** a. being a BSC student; b. being a resident of one of the five participating countries; and c. having access to online SNSs or similar platforms. The **exclusion criteria** were as follows**:** a. residing in a country not included in the study; and b. being students of the investigators/collaborators.

### Ethical considerations

This study sought approval from Assiut University in Egypt (**IRB 08/08/2017 number 38**) and ethical clearance in the respective participating countries. This study is a non-experimental study and did not utilize human subjects. It was performed by seeking permission and approval from the respective focal countries collaborating in this research. The three-part survey tool was administered through the use of an online survey, with a written consent section provided to proceed and to seek the respondents’ willingness to participate in the study. Returning the electronically tallied survey form indicated a willingness to participate. The identities of the participants and their personal information were left undisclosed. Blind tallying was used to secure privacy, and codes were used to maintain the anonymity of the participants. All respondents were informed that they could voluntarily withdraw from the study.

### Data gathering procedure

The main communication letter with the approval of the IRB was sought from the preidentified colleges and universities in the five participating countries mentioned above. Once approval from the IRBs in each research setting was obtained, the corresponding co-researchers were in charge of the selection of the study participants based on the inclusion and exclusion criteria. Data collection took place between spring 2017 and fall 2018. Through a hosting site, a web-based online tool was forwarded as a link to the study participants for easy access.

### Research instrument

The research instrument was subjected to both internal validity and reliability testing. Face validity and content validity were assessed and screened by two experts in the field of nursing research. A post hoc reliability test was performed, and the results of Cronbach’s α yielded a reliability of 0.92 and a margin of error of 0.8. A three-part questionnaire was utilized. Part 1 of the questionnaire sought to determine the demographic profile of the participants in terms of age, gender, the year level, the type of social media site used, and the country of residence. Part 2 of the questionnaire concerned the extent to which SNSs are utilized as a means of communication for educational purposes among nursing students. Finally, part 3 of the questionnaire addressed the perceived benefits of SNSs for nursing students. Both parts 2 and 3 used a four-point Likert scale. When responding to Likert-based questionnaire items, the respondents specified their level of agreement with a statement. They were asked to check the number that best corresponded to their answer regarding the extent of utilization and the perceived benefits of SNSs among nursing students. The highest score was 4, and the lowest score was 1.

### Data analysis

The results of this study were analysed and interpreted using the Statistical Package for the Social Sciences (IBM SPSS 24.0). The **weighted mean (**Table 1**and** Table 2**) was** used to determine the average extent of SNS utilization among nursing students. It was also used to determine the perceived benefits of SNSs among nursing students in terms of the accessibility, usability, efficiency, and reliability of SNSs. After gathering all the completed questionnaires, the mean was computed and gauged according to the following range and qualitative sinterpretations:

**Table 1:** Using a four-point Likert scale (extent of SNS utilization)

Weight	Mean Range	Adjectival Rating	Interpretation
4	3.51–4.50	Always	Highly utilized
3	2.51–3.50	Often	Moderately utilized
2	1.51–2.50	Sometimes	Slightly utilized
1	1.0–1.50	Never	Not utilized

**Table 2:** Using a four-point Likert scale (perceived benefits of SNSs)

Weight	Mean Range	Adjectival Rating	Interpretation
4	3.51–4.50	Always	Highly beneficial
3	2.51–3.50	Often	Moderately beneficial
2	1.51–2.50	Sometimes	Slightly beneficial
1	1.0–1.50	Never	Not beneficial

**Repeated-measures ANOVA** was also utilized to identify any significant differences between the two different mean domains, and a post hoc test was performed using **Bonferroni’s α** [20]. The **Mann-Whitney U test** was used to test two or more independent samples that were drawn from the same population where the level of measurement was ordinal [21]. **Pearson’s r** is both descriptive and inferential [20], and it was used to determine the magnitude and direction of a significant relationship between the extent of utilization and the perceived benefits of SNSs among nursing students and to determine the relationship between students’ demographic profile, SNS utilization and the perceived benefits of SNSs and the potential of SNSs to improve the participants’ study habits. The statistical power used for correlations is 1.

## Results

The study recruited 1200 participants, based on which a post hoc sample using proportion analysis yielded 1137 students who were taken as the actual sample for this study. The profile distribution of nursing students grouped by country showed that the students from Israel were mostly 26–28 years old, female and first-year students. The nursing students from Iraq were mostly 20–22 years old, female and second-year students. In Oman, most of the nursing students were also 20–22 years old and female, and they were not classified as being first- to fifth-year students. They were irregular students who could be placed in between year levels depending on their nursing major courses, and they could be clustered in a specific year. In the Philippines and Turkey, most of the students were 20–22 years old, female and third-year students. Overall, the majority of the students were 20–22 years old, female and third-year students **(**Table 3**)**.

**Table 3:** Participants Profile

Profile	Categories	Israel	Iraq	Oman	Philippines	Turkey	Total
**Age**	**16–19 yrs old**	5 (0.4%)	43(3.8%)	74(6.5%)	12(1.1%)	25(2.2%)	159(14.0%)
	**20–22 yrs old**	45 (4.0%)	151 (13.3%)	229 (20.1%)	51 (4.5%)	144 (12.7%)	620 (54.5%)
	**23–25 yrs old**	43 (3.8%)	59 (5.2%)	39 (3.4%)	9 (0.8%)	33 (2.9%)	183 (16.1%)
	**26–28 yrs old**	72 (6.3%)	11 (1.0%)	13 (1.1%)	3 (0.3%)	3 (0.3%)	102 (9%)
	**30 and above**	54 (4.7%)	10 (0.9%)	8 (0.7%)	0 (0%)	1 (0.1%)	73 (6.4%)
	**Total**	219 (19.3%)	274 (24.1%)	363 (31.9%)	75 (6.6%)	206 (18.1%)	1137 (100%)
**Gender**	**Male**	60 (5.3%)	119 (10.5%)	110 (9.7%)	14 (1.2%)	56 (4.9%)	359(31.6%)
	**Female**	159 (14%)	154(13.5%)	246(21.6%)	60(5.3%)	150(13.2%)	769 (67.6%)
	**Prefer not to say**	0 (0%)	1(0.1%)	7 (0.6%)	1(0.1%)	0 (0%)	9(0.8%)
	**Total**	219 (19.3%)	274 (24.1%)	363 (31.9%)	75 (6.6%)	206 (18.1%)	1137 (100%)
**Year Level**	**Year I**	63 (5.5%)	12 (1.1%)	0 (0%)	0 (0%)	0 (0%)	75 (6.6%)
	**Year II**	37 (3.3%)	117 (10.3%)	37 (3.3%)	0 (0%)	0 (0%)	191 (16.8%)
	**Year III**	48 (4.2%)	76 (6.4%)	77 (6.8%)	72 (6.3%)	163 (14.3%)	436(38.3%)
	**Year IV**	52 (4.6%)	35 (3.1%)	71 (6.2%)	0 (0%)	42 (3.7%)	200 (17.6%)
	**Year V**	0 (0%)	24(2.1%)	56(4.9%)	2(0.2%)	1(0%)	83(7.3%)
	**Others**	19(1.7)	10(0.9)	122 (10.7%)	1 (0%)	0 (0%)	227 (20)
	**Total**	**219 (19.3%)**	**274 (24.1%)**	**363 (31.9%)**	**75 (6.6%)**	**206 (18.1%)**	**1137 (100%)**

**Fig. 1:**
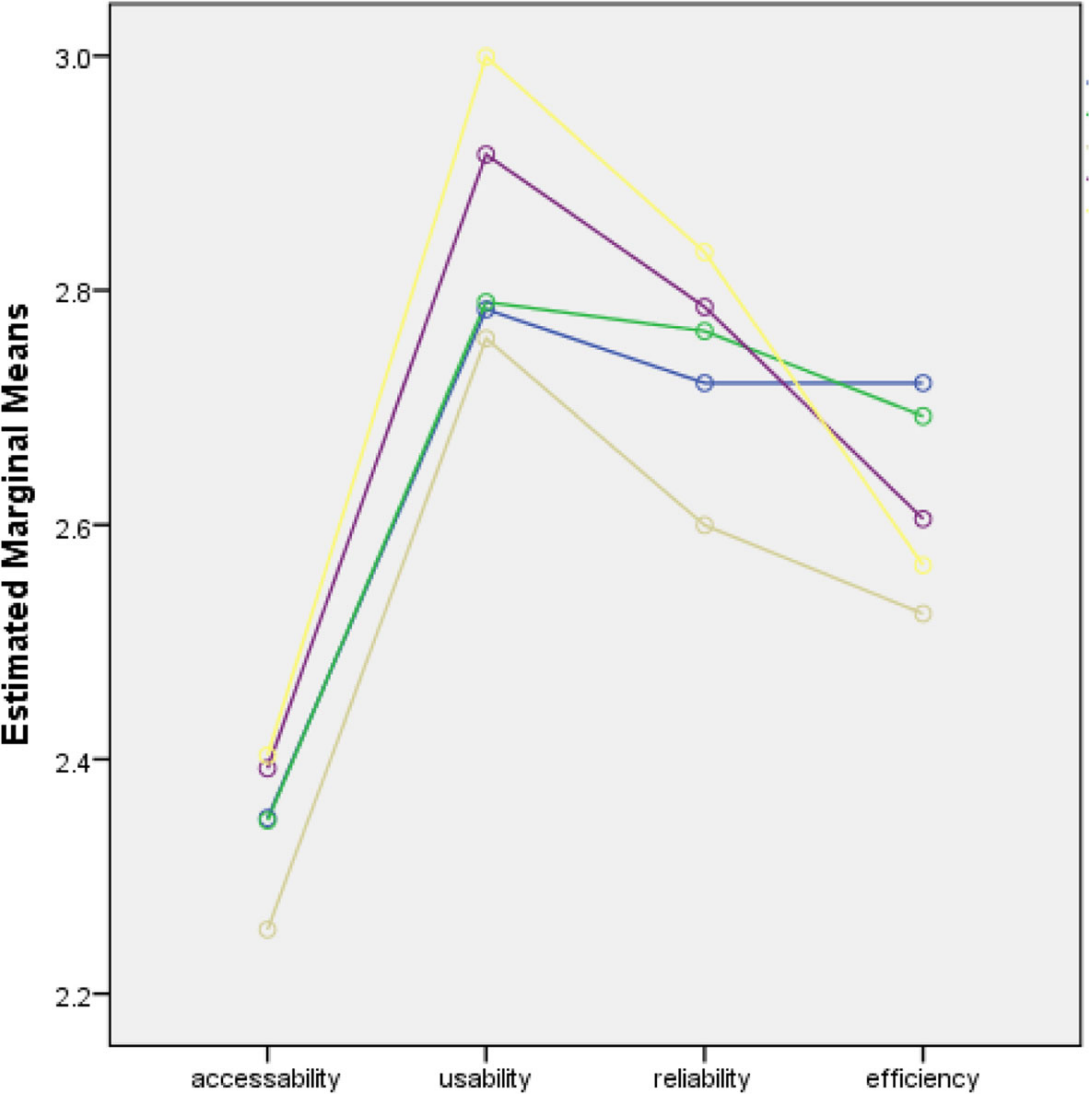
Line Chart of the Extent of Utilization of the Nursing Students Across All Domains when grouped by Age

**Fig. 2:**
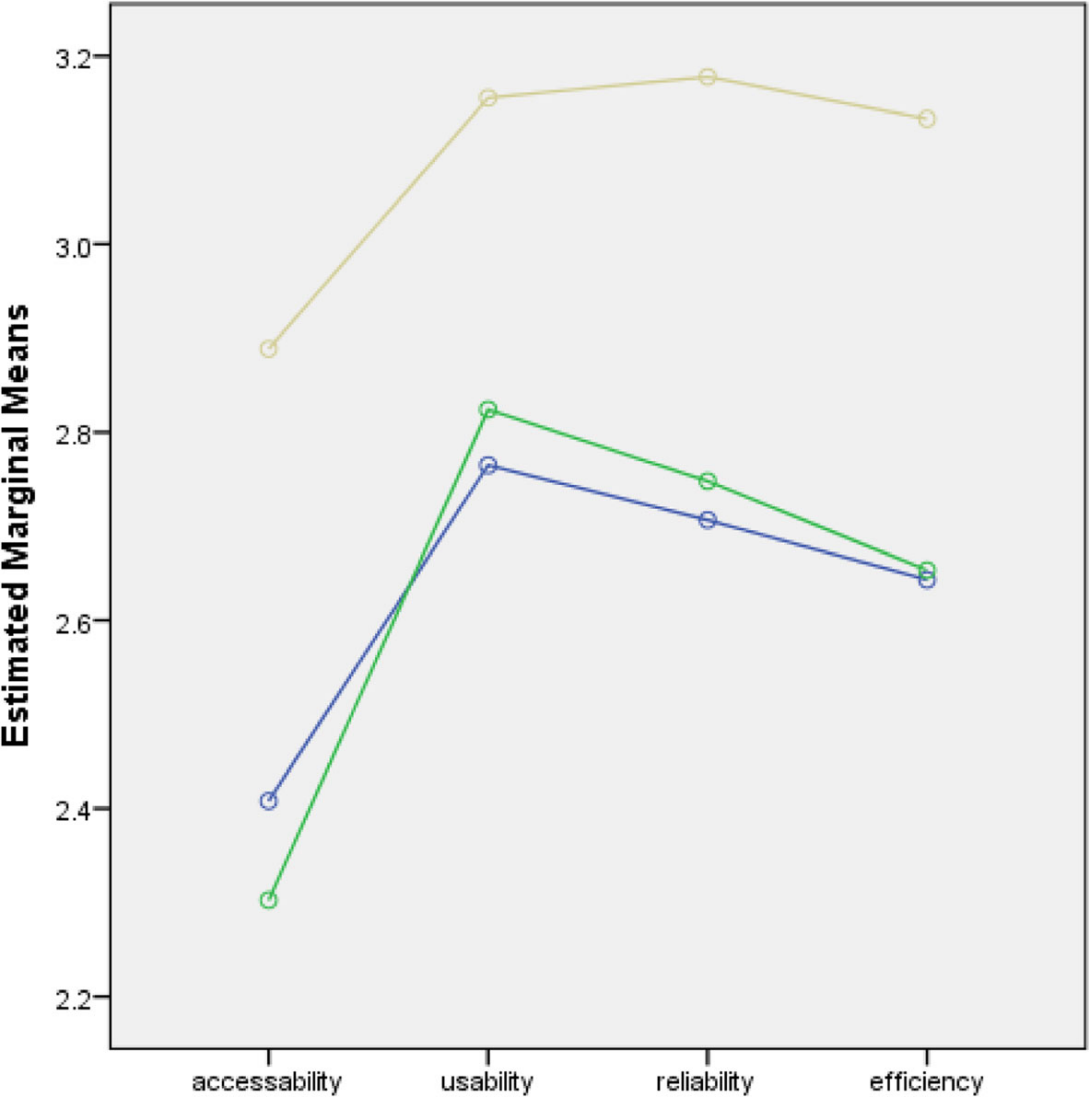
Line Chart of the Extent of Utilization of the Nursing Students Across All Domains when grouped by Gender

**Fig. 3:**
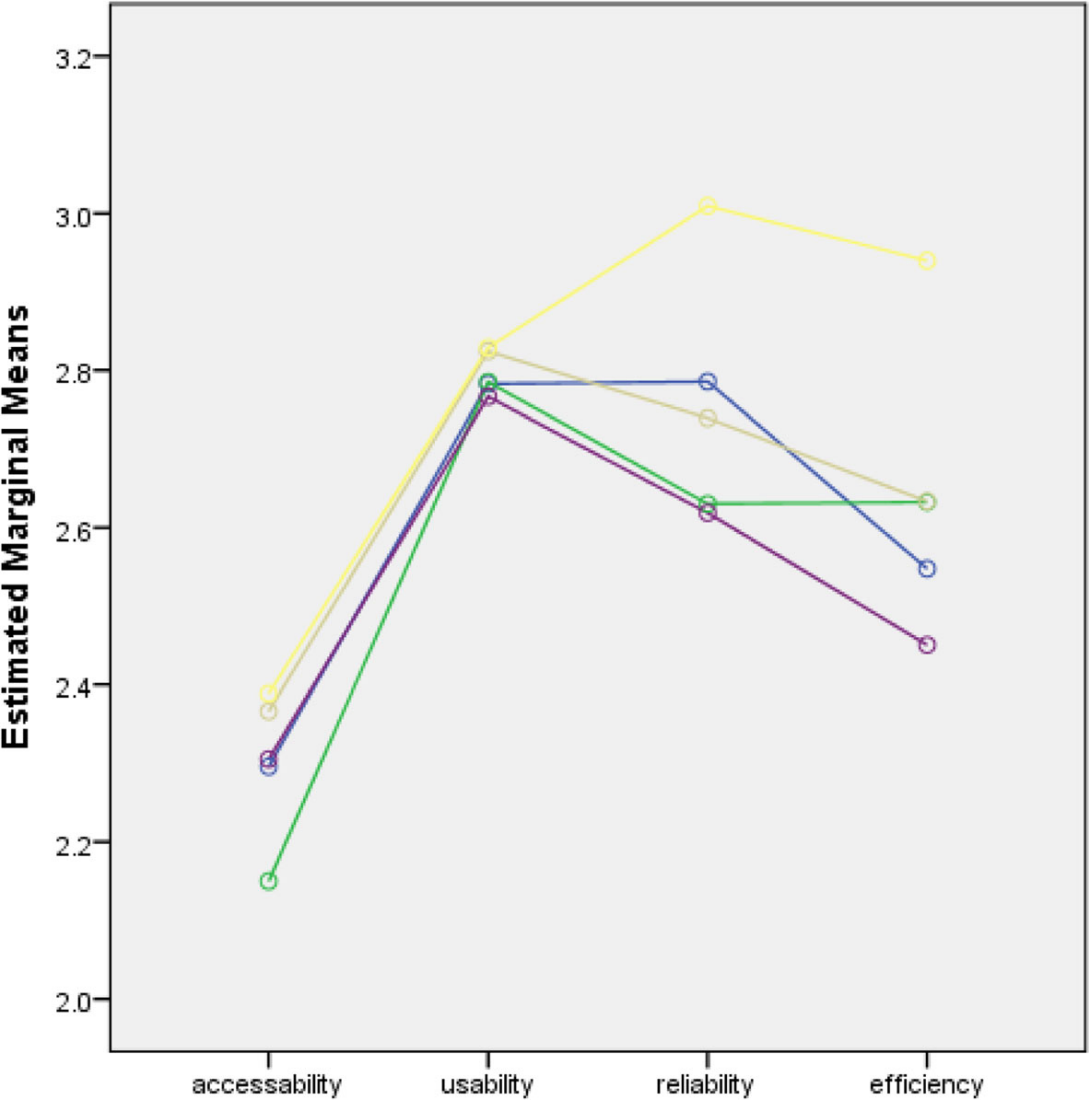
Line Chart of the Extent of Utilization of the Nursing Students Across All Domains when grouped by Year

**Fig. 4:**
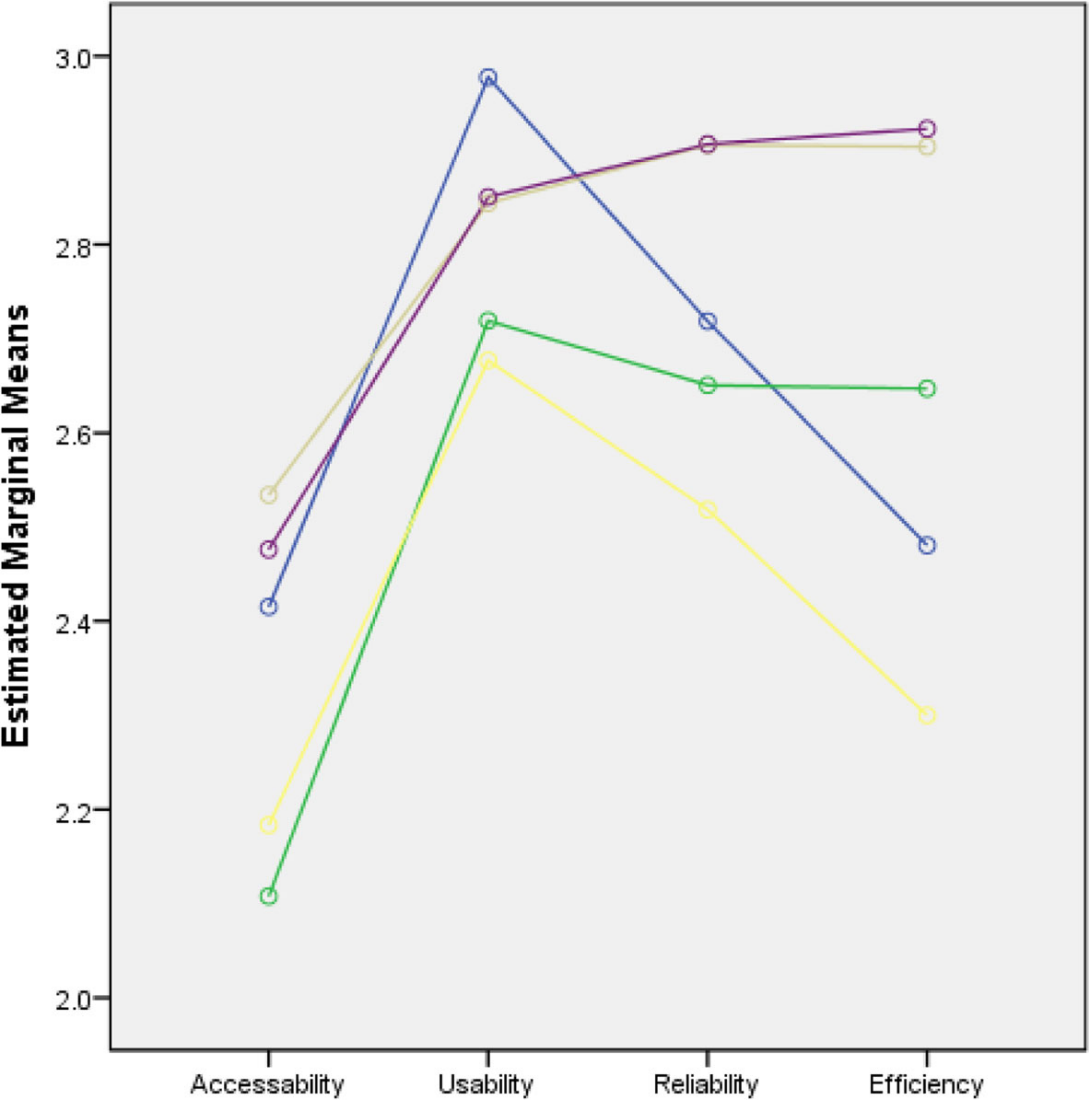
Line Chart of the Extent of Utilization of the Nursing Students Across All Domains when grouped by Country

The percentage distribution of the extent to which SNSs were utilized as a means of communication for educational purposes among nursing students in the five countries showed that the majority of nursing students slightly utilized SNSs in terms of their accessibility (61.3%) and moderately utilized them in terms of usability (60.2%). The distribution also showed that most of them moderately utilized SNSs in terms of their efficiency (45.2%) and reliability (46.8%) **(**Table 4**).** Figures 1, 2, 3 and 4 show the extent of SNS utilization among nursing students grouped according to age, gender, the year level and country. The results also revealed that nursing students had varied responses in terms of their perception of the extent to which SNSs were utilized as a means of communication. At least 2.1% and at most 6.2% of nursing students did not utilize SNSs, and 27.8 to 61.3% of nursing students slightly utilized SNSs. It was also observed that more than one-fourth (30.6%) to 60.2% of the students moderately utilized SNSs. At most 16.8% of students perceived SNSs as being highly utilized. Moreover, on average, nursing students slightly utilized SNSs in terms of accessibility (2.34) and moderately utilized them in terms of usability (2.81), efficiency (2.74) and reliability (2.66). Similarly, nursing students slightly utilized SNSs in terms of accessibility. Regarding the extent of accessibility, the results indicated that nursing students sometimes used an Internet café (2.33), their campus (1.94), malls (2.42), restaurants (2.12), game consoles (2.23), an iPad (1.76) or USB broadband (2.20). They often accessed SNSs in their own houses (2.88) and via mobile phones (2.52) and portable laptops (3.01). In terms of usability, nursing students moderately utilized SNSs. This result means that they often utilized SNSs to receive updates on school activities (3.10), to gain more knowledge about their current lessons (2.97), to share their thoughts and opinions about discussions (2.79) and to carry out advanced studies (2.74). Sometimes, they utilized SNSs for communication purposes related to their studies (2.40). In terms of reliability, the results revealed that they often relied on SNSs to familiarize themselves with their future lessons (2.71), to receive updates on school activities (2.69), to improve their knowledge and skills (2.79), to participate in group research (2.72) and to carry out assignments and projects (2.75). This result means that they moderately utilized SNSs. In terms of efficiency, nursing students often enhanced their abilities to provide nursing care through SNSs (2.82). They often considered that the sources obtained from SNSs were accurate (2.71) and that they learned proper techniques related to nursing skills by using SNSs (2.56) **(**Table 5**).** Nursing students were also recognized by their clinical instructors because of the expertise obtained from SNSs (2.39). This result meant that they moderately utilized SNSs.

**Table 4:** Frequency and Percent Distribution of the Accessibility, Usability, Efficiency and Reliability of the Extent of Utilization of Social Networking Sites as a means of Communication

Extent	Accessibility		Usability		Efficiency		Reliability	
	F	%	f	%	f	%	f	%
not utilized (NU)	71	6.2	24	2.1	55	4.8	45	4.0
slightly utilized (SU)	697	61.3	316	27.8	368	32.4	427	37.6
moderately utilized (MU)	348	30.6	684	60.2	514	45.2	532	46.8
highly utilized (HU)	21	1.8	107	9.4	191	16.8	129	11.3
No data	0	0	6	0.5	9	0.8	4	0.4
**Mean (QD)** **SD**	**2.34 (SU)** **0.529**		**2.81 (MU)** **0.612**		**2.74 (MU)** **0.707**		**2.66 (MU)** **0.637**	

*n* = 1137

**Table 5:** Means, Standard Deviations and Qualitative Description of the Utilization of Social Networking Sites

Statements	Mean	Std. Deviation	Qualitative Description
1. I work on my assignments using my own computer inside our house.	2.88	1.030	Often
2. I go to internet cafes near our house to do my assignments.	2.33	1.240	Sometimes
3. I utilize social networking sites inside the campus to help me on my seatwork.	1.94	1.026	Sometimes
4. I utilize social networking sites in malls to get me updated on school activities.	2.42	1.058	Sometimes
5. I can access social networking sites in restaurants for research purposes.	2.12	1.018	Sometimes
6. I can access social networking sites for my assignments through my mobile phone.	2.52	1.115	Often
7. I can access social networking sites for my assignments through my portable laptop.	3.01	1.014	Often
8. I can access social networking sites for my assignments through game consoles like Playstation3 and Xbox360.	2.23	1.206	Sometimes
9. I can access social networking sites for my assignments through my iPad.	1.76	1.037	Sometimes
10. I can access social networking sites for my assignments through my USB broadband.	2.20	1.090	Sometimes
Accessibility	2.34	.529	Slightly utilized
1. I utilize social networking sites for communication purposes in relation to my studies.	2.40	1.030	Sometimes
2. I utilize social networking sites to get me updated in school activities.	3.10	.869	Often
3. I utilize social networking sites to gain more knowledge about my current lessons	2.97	.888	Often
4. I utilize social networking sites to share my thoughts and opinion about our discussions.	2.79	.995	Often
5. I utilize social networking sites for advance studies.	2.74	.884	Often
Usability	2.81	.612	Moderately Utilized
1. I rely on social networking sites to familiarize myself with future lessons.	2.71	.988	Often
2. I rely on social networking sites to get me updated on school activities	2.69	.864	Often
3. I rely on social networking sites to improve my knowledge and skills.	2.79	.973	Often
4. I rely on social networking sites for group researches.	2.72	.865	Often
5. I rely on social networking sites for my assignments and projects.	2.75	.946	Often
Reliability	2.74	.707	Moderately utilized
1. Social networking sites provide correct data and information	2.80	.916	Often
2. Social networking sites enhance my abilities in providing nursing care.	2.82	.866	Often
3. Sources from social networking sites are accurate.	2.71	.802	Often
4. I learn proper techniques related to nursing skills from social networking sites.	2.56	.875	Often
5. Clinical instructors recognize my expertise obtained from social networking sites.	2.39	.921	Sometimes
Efficiency	2.66	.637	Moderately utilized

Legend: 1.00–1.49 (never/not utilized), 1.50–2.49 (sometimes/slightly utilized), 2.50–3.49 (often/moderately utilized), 3.50–4.00 (always/highly utilized)

Regarding the question of what SNS nursing students found to be the most helpful, slightly more than one-fourth of nursing students considered Facebook (25.3%), WhatsApp (26%), and Google (25.8%) to be the most helpful social media networks. The results also showed that some students considered Instagram, Snapchat, e-learning, YouTube, Twitter, and others to be the most helpful. Three of the students (0.3%) claimed that they used no social media networks **(**Table 6**).** In terms of usability, reliability, accessibility, and efficiency, the results showed that nursing students perceived SNSs as slightly beneficial in terms of accessibility (2.34). They also revealed that SNSs were moderately beneficial in terms of usability, reliability, and efficiency.

**Table 6:** Frequency Count and Percent Distribution of the Most Helpful among Nursing Students

Social Media Network	Frequency	Percent
None	3	0.3
Facebook	288	25.3
Instagram	60	5.3
Whatsapp	296	26.0
Snapchat	41	3.6
Google	293	25.8
e-learning sites	23	2.0
Youtube	85	7.5
Twitter	34	3.0
Others	14	1.2
Total	1137	100.0

With regard to study habits, nursing students often have different study habits in terms of their time management, study focus, and personal perceptions of learning, as well as receiving good grades and carrying out assignments, in addition to the importance of earning exceptional grades. In terms of time management, students allotted enough time (2.85) for studying (2.74), scheduled a fixed time (2.94), and set the best time so that they could study (2.84), reviewing either every day (2.71) or every week (2.51). They also often considered how to focus entirely on studies (2.87) or how to become interested in their studies (2.93), for example, by seeking a quiet place (3.12) or, sometimes, by studying with music or while watching TV (2.41). Moreover, they often considered studying even without exams (2.70) or completing difficult assignments (2.70). They normally enjoyed learning (2.81), and they were always confident that they could receive good grades (3.10). They also frequently attached importance to earning exceptional grades (3), and they ensured that they knew which homework assignments to carry out (3.10) **(**Table 7**)**. The results of the extent of SNS utilization in terms of accessibility, usability, and reliability suggested that the younger the age group of the nursing students, the lower their extent of utilization, except for the 23–25 age group. However, the results of the extent of SNS utilization in terms of efficiency contradicted this possible correlation; it suggested that the younger the age of the students was, the lower the extent of SNS utilization in this area, except for the 23–25 age group. The results further showed that there was a significant difference in the extent of SNS utilization in terms of usability (χ2(4) = 16.038, *p* = 0.003) and efficiency (χ2(4) = 12.360, *p* = 0.015). There was also a significant result in terms of reliability (χ2(4) = 11.012, *p* = 0.026). However, pairwise comparison disconfirmed the result of a significant difference. The extent of SNS utilization in all areas was consistently higher in female nursing students, except for accessibility. This suggested a possible relationship where female students tended to have a higher extent of SNS utilization but not in terms of accessibility. The Mann-Whitney U test was performed, revealing that there was a significant difference in the extent of SNS utilization only in terms of accessibility. This result indicated that the extent of nursing students’ SNS utilization in terms of accessibility was significantly higher in male students than in female students. Since the results indicated a non-significant *p*-value (*p* > 0.05), this also meant that the extent of nursing students’ SNS utilization in terms of usability (*p* = 0.134), reliability (*p* = 0.264) and efficiency (*p* = 0.586) was the same regardless of gender. Regarding accessibility, fifth-year nursing students had the highest SNS utilization in terms of accessibility (Mn rank = 538.86), reliability (Mn rank = 603.22), and efficiency (Mn rank = 631.38). Fourth-year nursing students consistently had the lowest extent of SNS utilization in terms of usability (Mn rank = 471.68), reliability (Mn rank = 448.22), and efficiency (Mn rank = 419.48) but not accessibility (Mn rank = 486.23). It was also observed that there was a fluctuating pattern as the students’ year level increased, which was consistent with the results presented.

**Table 7:** Means, Standard Deviations, and Qualitative Descriptions of Extent of Doing Possible Study Habits

	Mean	Std. Deviation	Qualitative Description
1. Do I allot specific number of hours for studying?	2.74	.944	Often
2. Do I follow a definite time schedule?	2.94	.903	Often
3. Do I take time to study everyday	2.71	.942	Often
4. Do I know which time of the day I can study best?	2.84	.962	Often
5. Do I easily find enough time to study?	2.85	.959	Often
6. Do I allot time every week to review?	2.51	.910	Often
7. Am I able to focus entirely when I study?	2.80	.875	Often
8. Do I get interested on my studies?	2.93	.824	Often
9. Do I easily concentrate when I study?	2.87	.800	Often
10. Do I usually seek a quiet place to study?	3.12	.788	Often
11. Am I able to study best with music on/ while watching TV?	2.41	1.038	Sometimes
12. Do I study even when there are no quizzes and exams?	2.50	1.037	Often
13. Do I easily complete a difficult assignment?	2.70	.889	Often
14. Do I enjoy learning?	2.81	.822	Often
15. Do I believe I could get better grades?	3.10	.852	Often
16. Before I leave class, do I make sure that I know which homework to accomplish	3.10	.847	Often
17. Are exceptional grades important to me?	3.00	.843	Often
18. Do I prioritize studying over other activities?	2.95	.860	Often
19. Do I exert extra effort when I study?	2.98	.812	Often

Legend: 1.00–1.49 (never), 1.50–2.49 (sometimes), 2.50–3.49 (often), 3.50–4.00 (always)

From the initial extent of SNS utilization of first-year nursing students, the extent of SNS utilization of second-year students was lower compared to that of first-year students. The extent of SNS utilization was higher in third-year students than in fourth-year students. Additionally, the extent of SNS utilization among fourth-year students was lower than that among fifth-year students. Inferential testing was performed through the Kruskal-Wallis test. The results of the test revealed that there were significant differences in the extent of SNS utilization in terms of accessibility when grouped by the year level (χ2(4) = 19.897, *p* = 0.001), reliability (χ2(4) = 21.345, *p* < 0.01), and efficiency (χ2(4) = 33.682, *p* < 0.01). However, no significant difference in the extent of SNS utilization in terms of usability was found (χ2(4) = 1.187, *p* = 0.880). A significant difference was found between the extent of utilization and the perceived benefits of SNSs in terms of accessibility (χ2(4) = 126.981, *p* < 0.01), usability (χ2(4) = 40.096, *p* < 0.01), reliability (χ2(4) = 51.915, *p* < 0.01), and efficiency (χ2(4) = 147.964, *p* < 0.01) **(**Table 8**)**. It was observed that Oman and the Philippines had the highest mean ranks among all five countries, except for SNS utilization in terms of usability (where Israel obtained the highest mean rank). This result indicated that nursing students in Oman had the highest SNS utilization in terms of accessibility and reliability. The Philippines had the highest SNS utilization in terms of reliability but with a slight difference compared with Oman. Moreover, Turkey obtained the lowest mean rank in all areas, except in terms of accessibility. This result indicated that Turkey had the lowest SNS utilization in terms of usability, reliability, and efficiency. The extent of SNS utilization by nursing students was the highest in terms of usability (2.81), followed by reliability (2.74), efficiency (2.65) and accessibility (2.34) **(**Table 9**)**.

**Table 8:** Inferential Results on Significant Difference in the Extent of Utilization and Perceived Benefits of Social Networking Sites among Nursing Students when grouped by Profile Variables

Grouping Variable	Accessibility	Usability	Reliability	Efficiency
Age	Χ^2^(4) = 6.89 *p* = 0.142	Χ^2^(4) = 16.04 *p* = 0.003**	Χ^2^(4) = 11.01 *p* = 0.026*^a^	Χ^2^(4) = 12.36 *p* = 0.015*
Gender	U = 122,944 *p* = 0.003**	U = 129,223 *P* = 0.134	U = 130,415 *P* = 0.264	U = 134,163 *P* = 0.586
Year Level	Χ^2^(4) = 19.90 *p* = 0.001**	Χ^2^(4) = 1.187 *p* = 0.880	Χ^2^(4) = 21.**35***p* = 0.000**	Χ^2^(4) = 33.68 *p* = 0.000**
Country	Χ^2^(4) = 126.98 *p* = 0.000**	Χ^2^(4) = 40.10, *p* = 0.880	Χ^2^(4) = 51.92, *p* = 0.000**	Χ^2^(4) = 147.96, *p* = 0.000**

*significant at 0.05 **significant at 0.01

^a^pairwise comparison disconfirmed that sig. Diff. exist

**Table 9:** Repeated Measures ANOVA Result on Significant Difference among each domains of the extent of utilization and perceived benefits of social networking sites among nursing students

factor1	Mean	Std. Error	F	df	Sig.
Accessibility	2.34	.017	151.334	3	.000^a^
Usability	2.81	.021			
Reliability	2.74	.024			
Efficiency	2.65	.020			

^a^significant at 0.01 *significant at 0.05

Furthermore, the results of repeated-measures ANOVA revealed that there was a significant difference among the domains of SNS utilization. Hence, in an additional test performed using Bonferroni’s post hoc test, accessibility was significantly lower than usability, reliability or efficiency. However, usability was significantly higher than reliability and efficiency, and reliability was significantly higher than efficiency **(**Table 10**)**. Pearson’s r revealed a significant positive correlation between the extent of a possible improvement in study habits and the extent of SNS utilization in terms of the four domains, namely, accessibility (r = 0.246), usability (r = 0.377), reliability (r = 0.287) and efficiency (r = 0.387). This result meant that there was a direct relationship between the two variables and further meant that the more the nursing students studied, the higher the extent of their SNS utilization in terms of accessibility, usability, reliability, and efficiency **(**Table 11**)**.

**Table 10:** Bonferroni Result

(I) factor1	(J) factor1	Mean Difference (I-J)	Std. Error	Sig.
Accessibility	Usability	−.470	.022	.000^a^
	Reliability	−.397	.025	.000^a^
	Efficiency	−.308	.023	.000^a^
Usability	Reliability	.074	.019	.001^a^
	Efficiency	.163	.021	.000^a^
Reliability	Efficiency	.089	.019	.000^a^

^a^significant at 0.01 *significant at 0.05

**Table 11:** Pearson-r Result on Relationship between the Extent of Doing Possible Study Habits and Extent of Utilization of Social Networking Sites

		accessibility	usability	reliability	efficiency
potential	Pearson Correlation	.246^a^	.377^a^	.287^a^	.387^a^
	Sig. (2-tailed)	.000	.000	.000	.000
	N	1134	1130	1128	1133

^a^Correlation is significant at the 0.01 level (2-tailed)

## Discussion

The findings of this study identified SNSs and the relationship between their utilization, their perceived benefits and their potential for improving the study habits of nursing students in five different countries. Based on the analysis of the findings of this study, most student respondents were 20–22 years old, female, and in their third year. Our findings are similar to those of a study conducted in Pakistan, where the majority of the nursing respondents were female and within the 21–25 age group [22]. A relevant finding explained how social media are an important aspect of today’s adolescents, offering efficiency if properly utilized [23]. A similar study on social networking identified that SNS addiction was higher in male than in female students [24].

This study revealed that the majority of the nursing students across the five countries were more engaged in websites and SNSs, such as Facebook, WhatsApp and Google. A study conducted in 2009 in Brazil and Singapore showed the wide utilization of Facebook on a regular basis [25]. These findings were also obtained in earlier studies where Myspace and Facebook were among the most popular sites among students, even though they were not created for educational purposes [26]. In the results of this study, it was also evident that the use of SNSs was important for establishing communication for educational purposes, and 61.3% of the respondents utilized SNSs for the purpose of relaying information relevant to their studies.

A study has suggested that SNSs are platforms that can be used to improve educational impacts by adapting modifications in the instructional curricula of medical schools [2]. The aspect of accessibility is an important factor in today’s generation of Internet-savvy students, and the study findings suggest the great importance of accessibility. It was found that students were able to gain access to their social networking profiles through Internet cafés, malls, restaurants and their campus. A study mentioned that access to information was just a click away and that the accessibility of the information on the Internet and SNSs was widely used, which was inherently identified as the main reason why most students were no longer visiting libraries [27]. Most students prefer SNSs because of their quick and easy access and, in particular, for the purpose of education and learning.

The usability of SNSs in terms of educational purposes is a topic that needs contextualization, as the study findings showed that nursing students in the five countries use SNSs for educational gains by taking advantage of the Internet to acquire knowledge on current lessons, by receiving updates on ongoing school activities, and by carrying out advanced studies. Many educational institutions are still dependent on a traditional learning system, which does not use the full capacity of SNSs as a tool for teaching and learning [28]. The results of this study contradict those of a study conducted in Oman, where the findings showed that SNSs were mainly used for entertainment purposes and were less used for educational purposes [29]. SNSs can present various media, such as photos, videos, interactive interfaces and games, which make them highly engaging among students. Moreover, nursing students engage in more interactive skill-based learning sessions. In terms of reliability, nursing students from the five participating countries identified that SNSs were moderately utilized for the purpose of keeping track of school activities and improving knowledge and skills. Regarding efficiency, students scored high in providing correct data and information, enhanced their abilities to provide nursing care, and learned how to perform proper techniques relevant to their nursing skills. It was also noted that some clinical instructors recognized the expertise of students drawn from SNSs, which was supported by a study intervention using SNSs that taught nursing students about ethical and moral behaviours through humanized mannequins in social networks, such as Facebook [30].

Advanced teaching strategies and the availability of updated and timely learning materials can be advantageous as learning platforms for nursing students. Overall, the nursing students in all five countries were aligned in that they moderately utilized SNSs. In terms of benefits, the students from the five countries said that SNSs were highly beneficial. According to a study, 54.92% of dental students at a university in India suggested that the usage of SNSs was beneficial for their studies and learning needs [31]. This result is supported by an online survey on social networking as a learning tool that found that the majority of students perceived SNSs as an innovative method of study support that guided learning and enhanced efficacy [17]. However, the results of this study contradict study results on the effects of online social networking on student performance that suggest that the time that medical students spend on SNSs could negatively influence their academic achievement [32]. The negative and positive aspects of SNS utilization are a contentious issue that has yet to be resolved because SNSs can be addictive and their improper usage may lead to less positive outcomes. Studying is a skill, and developing study habits is vital for the academic performance of students [33]. Some studies strongly advocate the use of SNSs as a means of becoming academically successful. For example, one study mentioned that Facebook and SNSs were considered the greatest distractions among college students, subsequently affecting their study habits and grades [34]. Based on the perspectives of nursing students with regard to their study habits, the study participants from the five countries unanimously identified time management as essential, and a fixed schedule was important when utilizing social networking platforms. This was evidently described by the results of a study showing that SNSs could enhance performance in a simple task environment but made no difference in a complex performance environment [35]. SNS utilization was also found to be consistently high among female nursing students. It is a known fact that nursing is female dominated [36]; there are confirmed gender differences that exist with regard to the technologies adopted, and they occur between genders from the age of 16 to 35 [37]. These findings are firmly contradicted by a study conducted in China showing that Chinese females were clearly less engaged with technology than Chinese males [38]. On the other hand, women who were found to have higher introversion and extraversion traits turn to the Internet for social services, such as online chats and discussion groups [39].

In a geographical and cultural context, it can be seen that in countries such as Iran, Israel, Oman and Turkey, the female gender is given less opportunity for public exposure, which results in a higher use of SNSs, which are viewed as a viable medium to socialize and be engaged with others instead of being physically present. A study observed that cultural considerations influenced the interaction platform of choice and the use of SNSs [40]. Oman and the Philippines were identified as having the highest SNS utilization. In a study of health science students conducted by Sultan Qaboos University, the findings showed that YouTube, Facebook, and Twitter were the most commonly used social media platforms. The findings generally suggest that usage and addiction are similar worldwide [41]. On the other hand, in the Philippines, the US-based Pew Research Center said that 88% of Filipinos felt that increasing Internet usage was good for education, given that the Philippines is often dubbed the “social media capital” of the world [42]. In contrast, with regard to SNS utilization, Turkey ranks lowest according to the findings of Kirschner and Karpinski in Turkey, whose study among undergraduate students revealed that students who reported academic problems were more likely to use the Internet for social networking (e.g., Facebook) purposes [43]. The results of the hypothesis testing yielded a positive relationship between study habits and the extent of SNS utilization among nursing students in the five participating countries. The levels of nursing students’ engagement in SNS utilization can be most beneficial and relevant when they uses SNS for purposes of studying. SNSs are deemed necessary in this generation of learners, wherein a significant amount of information is within grasp and readily available. The utilization of SNSs for educational purposes has both positive and negative implications [44, 45].

### Limitations

Our study has several limitations. Due to the cross-sectional nature of the study, it was not possible to explain the causal relationship with students’ demographic profile, such as their geographic location and culture, which will require a more extensive research design and strategy. In addition, the researchers acknowledge the lack of attention paid to the role of faculty members in facilitating the utilization of SNSs among nursing students in the selected countries.

## Conclusion

The paucity of research and policies related to the integration of SNSs as a learning tool requires attention from both researchers and policymakers. The nursing students from the five participating countries were female dominated, and the extent of SNS utilization was higher among females. This study also identified that the nursing students moderately perceived the utilization and benefits of SNSs, taking into account accessibility, usability, efficiency and reliability. The most commonly utilized social media platforms in Israel, Iraq, Oman, the Philippines, and Turkey were WhatsApp and Facebook. Regarding the correlations with utilization, perceived benefits and study habits showed a positive relationship among the three factors. Similarly, the significant positive correlation between the study habits of students and the extent of SNS utilization means that the more students devote themselves to their study habits, the higher the level of SNS utilization.

### Recommendations

This study further suggests that similar studies in the future should focus not only on the aspects of access, usability, efficiency and reliability but also on the inclusion of behavioural aspects. Cultural differences can also be taken into consideration. The homogeneity of the sample can also be addressed by tapping more diverse nursing student populations. Four out of five participating countries (Israel, Iraq. Oman and Turkey, with the Philippines being the exception) are homogenous in terms of culture and geographic settings. A mixed-method approach in future studies is also recommended to contextualize the confounding influence of culture and geographic location. Although there are several studies on SNSs and academic performance, very few studies in nursing academia have been conducted that focus on skills or psychomotor development through virtual platforms that can also be used in the teaching-learning process. The influences of SNSs on nursing students and their great potential for enhancing the study habits of students are an area of opportunity in regard to developing curricula that are not restricted to the four corners of the classroom. SNSs are by far the most current and the most relevant platforms that can further add to the learning success and academic achievement of nursing students. Tailored strategies for enhancing student participation, interaction and real-life learning are just a few of the advantages that can be obtained by tapping the positive contributions of SNSs as a teaching-learning tool in nursing education.

## Data Availability

All data generated or analysed during the current study are available from the corresponding author upon reasonable request.

## Abbreviations

SNS: Social networking site
BSC: Bachelor of Science in Nursing
IRB: Institutional review board
MARG: Mentor and researcher group
ICT: Information and communication technology
SPSS: Statistical Package for the Social Sciences
